# Influence of environmental information in natural scenes and the effects of motion adaptation on a fly motion-sensitive neuron during simulated flight

**DOI:** 10.1242/bio.20149449

**Published:** 2014-12-12

**Authors:** Thomas W. Ullrich, Roland Kern, Martin Egelhaaf

**Affiliations:** 1 Department of Neurobiology, Bielefeld University, Universitätsstrasse 25, 33615 Bielefeld, Germany; 2 Center of Excellence Cognitive Interaction Technology (CITEC), Bielefeld University, Inspiration 1/Zehlendorfer Damm 201, 33619 Bielefeld, Germany

**Keywords:** Adaptation, Fly, Contrast, Natural images, Nearness, Neural activity, Spatial vision

## Abstract

Gaining information about the spatial layout of natural scenes is a challenging task that flies need to solve, especially when moving at high velocities. A group of motion sensitive cells in the lobula plate of flies is supposed to represent information about self-motion as well as the environment. Relevant environmental features might be the nearness of structures, influencing retinal velocity during translational self-motion, and the brightness contrast. We recorded the responses of the H1 cell, an individually identifiable lobula plate tangential cell, during stimulation with image sequences, simulating translational motion through natural sceneries with a variety of differing depth structures. A correlation was found between the average nearness of environmental structures within large parts of the cell's receptive field and its response across a variety of scenes, but no correlation was found between the brightness contrast of the stimuli and the cell response. As a consequence of motion adaptation resulting from repeated translation through the environment, the time-dependent response modulations induced by the spatial structure of the environment were increased relatively to the background activity of the cell. These results support the hypothesis that some lobula plate tangential cells do not only serve as sensors of self-motion, but also as a part of a neural system that processes information about the spatial layout of natural scenes.

## INTRODUCTION

For decades, motion sensitive visual interneurons, like the lobula plate tangential cells (LPTCs) of flies, have been serving as a model system to study how motion information is processed and how orientation behavior is guided (Hausen, 1981; Hausen, 1984; Krapp et al., 2001; Egelhaaf, 2006; Borst et al., 2010; Borst and Euler, 2011; Egelhaaf et al., 2012). Most previous studies used experimenter-defined stimuli to characterize LPTCs, such as bars or grating patterns moving, for instance, at constant velocities or with a white noise velocity profile. Such stimuli are particularly suitable for a systems analysis of motion computation. On their basis, the motion signals of LPTCs were shown to depend not only on stimulus velocity and motion direction, but also on the brightness contrast, the spatial frequency and other properties of the stimulus pattern (Dvorak et al., 1980; Hausen, 1981; Egelhaaf and Borst, 1989; Egelhaaf et al., 1989; Hausen and Egelhaaf, 1989). However, these experimenter-defined stimuli differ much from what flies see, while behaving in their natural habitats. Therefore, it is not easily possible to infer the functional significance of LPTCs solely on this basis.

The visual input received by the eyes when moving in natural environments is not only characterized by the velocity and direction of self-motion, but also by the specific three-dimensional structure as well as textural features of the environment (van der Schaaf and van Hateren, 1996; Bex and Makous, 2002; Geisler, 2008). It is rather difficult to predict the responses of LPTCs to such natural image sequences from the knowledge obtained from experiments with simple stimuli, as the motion responses jointly depend in a nonlinear way on the velocity, the contrast and the spatial frequency content of the retinal images of the scenery (reviewed by Egelhaaf and Borst, 1993). Therefore, it is necessary to examine the responses of LPTCs to natural motion stimuli to be able to infer the potential functional role of LPTCs for visually guided orientation behavior. The optimal solution would be to record neural signals in animals behaving in their natural environment. As this is not possible for methodological reasons, alternative approaches have been employed to approximate different aspects of natural optic flow.

Previous studies emphasized the importance of rotational motion input and referred to the assumed function of LPTCs as detectors for self-rotation (Krapp and Hengstenberg, 1996; Krapp et al., 1998; Krapp et al., 2001). Accordingly, follow-up studies used panoramic images of natural sceneries rotating at constant velocities around the animal for stimulation in electrophysiological experiments (Dror et al., 2001; Shoemaker et al., 2005; Straw et al., 2008; Barnett et al., 2010; O'Carroll et al., 2011; Meyer et al., 2011). However, pure rotational image motion neglects the spatial structure of the environment as an important determinant of the natural retinal image flow, since only the retinal image motion induced during translational self-motion depends on the distance to objects and their position relative to the motion direction (Koenderink, 1986). This is likely to be of special functional interest, since flies separate the translational from the rotational motion component during their flights: short phases of rapid rotational self-motion, so-called saccades, alternate with intersaccadic phases of nearly pure translational motion (Land, 1973; Schilstra and van Hateren, 1999; van Hateren and Schilstra, 1999; Tammero and Dickinson, 2002; Boeddeker et al., 2005; Braun et al., 2010; Kern et al., 2012). This characteristic feature of insect flight has been interpreted as a strategy to facilitate the gathering of spatial information during intersaccadic intervals (Egelhaaf et al., 2012). Whereas the retinal velocities during saccades are very large and beyond the optimal velocity range of LPTCs, the intersaccadic translational optic flow is regulated and kept in the optimal velocity range by adjusting flight speed (Kern et al., 2012).

Some studies focused on the characteristic dynamics of the optic flow as experienced by flies during free-flight maneuvers (Kern et al., 2005; Boeddeker et al., 2005; Karmeier et al., 2006; Kern et al., 2006). Stimulus movies were presented, simulating the visual input experienced by flies, while flying in an experimental arena (Schilstra and van Hateren, 1999; van Hateren and Schilstra, 1999; Lindemann et al., 2003). In this way, LPTC responses during intersaccadic intervals were shown to provide information about the nearness of environmental structures, such as the walls of the flight arena or objects inserted close to the flight trajectory (Kern et al., 2005; Heitwerth et al., 2005; Karmeier et al., 2006; Liang et al., 2008; Liang et al., 2012). Moreover, the object-induced changes in the intersaccadic responses could be shown to further increase relatively to the cell's baseline activity as a consequence of motion adaptation (Liang et al., 2008; Liang et al., 2011). This finding obtained by way of behaviorally generated optic flow is in line with previous results with experimenter-defined motion stimuli: while maintained motion stimulation leads to a decrease of the cell response (Maddess and Laughlin, 1985; Harris et al., 2000; Kurtz et al., 2000), the response increments induced by brief changes in velocity, contrast and motion direction increase as a consequence of motion adaptation (Maddess and Laughlin, 1985; Kurtz et al., 2009). These results led to the conclusion that adaptational effects enhanced the influence of discontinuities in stimulus parameters and, thus, of features characterizing objects in the environment.

Although these conclusions were partly based on experiments performed with behaviorally generated motion sequences (Kern et al., 2005; Heitwerth et al., 2005; Karmeier et al., 2006; Liang et al., 2008; Liang et al., 2012), they were obtained in flight arenas with an artificial and simple spatial structure. Therefore, the present study examined for the first time how LPTC responses were affected by the spatial layout of natural cluttered environments. Specifically, we tested the hypothesis that the responses of LPTCs depend on the average nearness distribution of natural environments within the analyzed cell's receptive field and, thus, provide spatial information during simulated translations. We choose the H1 cell as an especially well-explored representative of LPTCs, because it can be recorded for a sufficiently long time to allow presentation of long image sequences (Hausen, 1976; Eckert, 1980; Krapp et al., 2001). Furthermore, we analyzed to what extent the neural responses were affected by the brightness contrast of the natural images. Finally, the study addresses the consequences of motion adaptation on LPTCs during stimulation with translational optic flow as experienced in natural cluttered environments.

## MATERIALS AND METHODS

### Visual stimulation

**Fig. 1.**
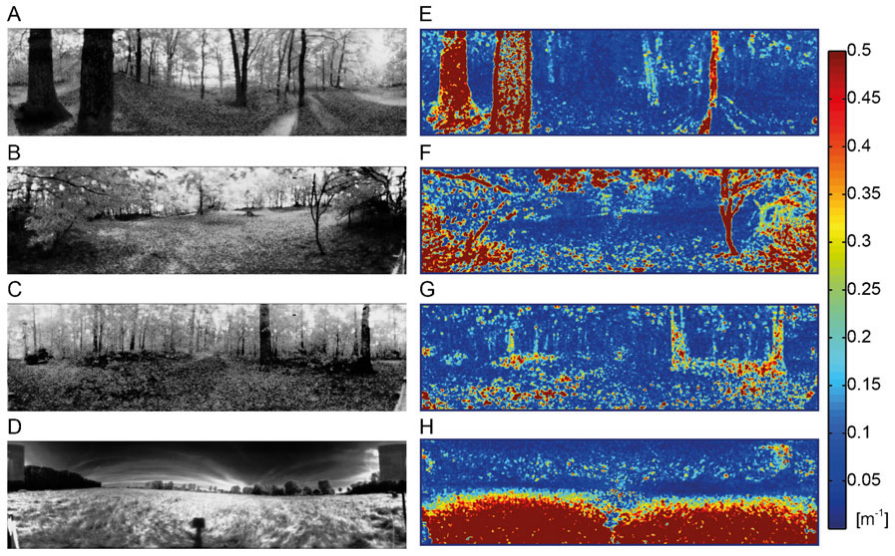
Examples of the natural images recorded in different natural environments and corresponding nearness maps. (A–D) Grayscale panoramic images of the natural scenes. Clearing in the forest with near trunks (A), near branches (B) and distant trees (C), open field (D). (E–H) Color-coded nearness maps of the same scenes.

The stimulus movies were generated from image series that had been recorded in a variety of natural environments in a parallel study (Schwegmann et al., 2014) (e.g. Fig. 1). Each image series consisted of a sequence of 100 panoramic images taken along a linear track with a length of 1 m in natural outdoor environments. By using a hyperbolic mirror on top of the camera the images covered 360° in azimuth and an elevation between −58° below and 47° above the horizon. The light was filtered by a dichroic filter that limited the camera's spectral sensitivity to a range of 480 to 550 nm, matching, to some extent, the spectral sensitivity of the visual motion pathway of flies (Stavenga, 2002). The camera sensor of 12-bit resolution had an adjustable gain, switching between two different values within one recording frame. In this way, a wide brightness range could be encoded by approximating a logarithmic sensitivity characteristic via two linear segments of different slope (‘LinLog-mode’). High dynamic range (HDR) images could, thus, be recorded with a single exposure, instead of calculating them from multiple images with different apertures or exposure times. The different image scenes were chosen to present stimuli with various depth distributions, e.g. near objects, like trunks of trees, or distant areas, like the edge of a forest beyond an open meadow. To present the HDR images by our stimulation device (see below) it was necessary to transform the 4096 distinct brightness values via a tone mapping function to the more limited dynamic range of just 256 distinct brightness values. Any kind of brightness transformation that could be used for this task reduces image information. Our requirements for the brightness transformation were: (1) A monotonic global relation between the original and the transformed brightness values. Thus, we excluded local tone mapping methods, as these can generate contrast artefacts. (2) Contrasts between adjacent pixels should be preserved, as such contrasts are important for the elementary motion detectors that feed the observed cell. This means that we tried to avoid large homogeneous areas, especially in the bottom area of the sceneries. (3) The overall brightness distribution should resemble the distributions found in the natural images.

Considering these requirements, we chose a global tone mapping method called ‘logarithmic histogram equalization’ (Larson et al., 1997), which is a reasonable trade-off between (2) and (3). A ‘simple histogram equalization’ strongly enhances contrasts and, thus, diminishes the loss of detailed information, but it also changes the overall brightness distribution, as each brightness bin gets apportioned to a similar number of pixels. However, in most natural scenes, the brightness distributions are uneven, with brightness values on the dark part of the scale being more abundant. By shaping the overall brightness distribution in a logarithmic way this feature is partly preserved. In a few control experiments, we tested this transformation against two other transformations: one was a ‘simple histogram equalization’ without a logarithmic shaping that preserves contrasts between pixels even better, but changes the shape of the brightness distribution considerably. The other was a ‘linear transformation’ of the original brightness by excluding the brightest 5% as well as the darkest 5% of the brightness values. In this case, the overall brightness distribution was preserved, whereas some more local contrasts were lost. A comparison between the neural responses to the motion sequences after the different transformations showed some differences in the absolute level of cell activation. However, considering the variability of the measured cell responses, there was no indication that these differences affect the conclusions drawn in the present study.

The stimulus movies were presented with the latest version of our custom-made stimulation device called FliMax (Lindemann et al., 2003). It allows a panoramic stimulation that covers large parts of the visual field of flies and is equipped with ultra-bright LEDs (maximal luminance ca. 12,000 cd/m^2^). We could show stimuli with a frame refreshing rate of 353.5 frames per second. The LEDs that represent the pixels of the stimulus movies are arranged on the surface of a partial icosahedron. The brightness values of the stimulus pixels for each frame were computed from the corresponding brightness level of the natural images. At the azimuth and the elevation of each pixel of the stimulus, the brightness values were averaged within a Gaussian window with a sigma value of about 2.4° being laid on a Lambert-azimuthal-equal-area projection of the image. This projection allowed us to minimize the distortion of the image, as it was centered for every pixel (for more details, see Schwegmann et al., 2014).

**Fig. 2.**
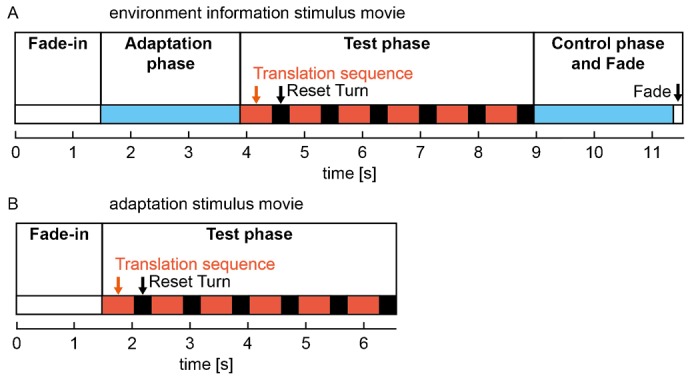
Outline of the two types of stimulus sequences employed in the analysis. (A) Environment information stimuli; (B) adaptation stimuli. The different sections of the stimuli are marked as colored blocks (periods of translational motion: red, accelerating/decelerating rotational motion: black, constant rotational motion: blue, no-motion: white).

We generated two types of motion stimuli based on the natural scenes: for examining the influence of the nearness and the brightness contrast of the natural scenery on the cell response we used the ‘environment information stimulus’ (Fig. 2A). This stimulus started with a fade-in to minimize effects of brightness changes and texture changes on the initial cell response: during the first second of stimulation the brightness of the screen changed from the mean brightness of the stimulus device to the mean brightness of the first image. In the following 0.5 s the first image of the image series faded in. To analyze the performance of the cell in its adapted state, we then started an adaptation phase by rotating the first image by 360° to the right within 2.4 s, which corresponds to an angular velocity of 150°/s. The following test phase consisted of six consecutive translation sequences simulating a backward translation corresponding to the preferred direction of the H1 cell, i.e. retinal image displacement from back to front. As the image series consisted of just 100 images, we interpolated intermediate images by averaging the brightness values of pairs of images consecutively recorded. Hence, we could present 199 images during each translation sequence. Each translation sequence lasted for approx. 560 ms, corresponding to a translational velocity of approx. 1.8 m/s. The translation sequences were followed by a reset turn setting the motion stimulus to the first image of the translation sequence. During the reset turn the pattern was rotated by 360° in the cell's preferred direction with a velocity profile similar to that of yaw saccades of flies (van Hateren and Schilstra, 1999). At the middle of the reset turn during the highest rotation velocity, the last image of the translation sequence was exchanged for the first image. After the sixth reset turn, a control phase equal to the adaptation phase followed. The stimulus movies ended with fading from the last natural image to the mean brightness of the stimulation device within 167 ms to match the first frame of the next stimulus movie.

The ‘adaptation stimulus’ (Fig. 2B) was similar to the ‘environment information stimulus’. However, to investigate how the process of motion adaptation affects the representation of environmental information during translatory motion, we changed the stimulus protocol in one important respect: to start the experiment with the cell being in its largely unadapted state, we omitted the adaptation phase as well as the corresponding ‘control phase’ and presented the test stimulus immediately after the fade-in. As no adapting rotational wide-field motion preceded the test phase, this experimental design allowed us to analyze how the effects of motion adaptation develop during successive stimulation with translational image sequences of natural scenes interspersed with reset turns akin to saccadic turns of the fly.

The responses of LPTCs differ depending on which of the different translational image sequences is shown, but variability also occurs across recordings of different cells. We used a characterizing stimulus to assess the response strength of individual cells which can then be used to normalize the cell responses when comparing the recordings from different cells. The first part of the characterizing stimulus was a vertical sinusoidal grating (wavelength 20°, Michelson contrast 0.98), horizontally moving at a temporal frequency of 1.92 Hz, first to the right and then to the left. Then a horizontal grating with the same properties moved upward and downward to ensure that no signals of neurons sensitive to vertical motion contribute to the overall response recorded.

Between the stimulus movies a stationary image was shown for 7 s to allow for recovery from motion adaptation. The stationary image was the last image of the previous stimulus. Hence, it was the mean brightness of the stimulus device in the case of the environmental information stimuli and the last natural image shown in the movie in the case of the adaptation stimuli. The stimuli were presented in a pseudorandom order. After having shown each stimulus twice, the characterizing stimulus was presented.

### Electrophysiology

For the experiments female blowflies of the species *Calliphora vicina* (Robineau-Desvoidy) were taken from our lab culture 3–5 days after hatching. We anaesthetized the flies with carbon dioxide and fixated them on a glass plate by using bees wax. We removed the legs and immobilized the proboscis and the antennae with bees wax. The head was bent forward and fixed to the thorax. The head capsule was opened at its back and some tracheae were removed to get free access to the lobula plate. The head of the fly was aligned to match the symmetry of the deep pseudopupil (Kirschfeld and Franceschini, 1968). Ringer solution (Kurtz et al., 2000) kept the tissue wet. The cell responses were recorded extracellularly from the output region of the left H1 cell in the right optic lobe, using sharp glass electrodes of borosilicate glass (G150TF-4, Warner Instruments, CT, USA). The electrodes were filled with 1M KCl solution. The H1 cell was characterized by its specific response profile: it is activated by wide-field back-to-front motion (preferred direction) contralaterally to our recording side (Hausen, 1981; Krapp et al., 2001). It responds with spikes to stimulation in its preferred direction, whereas motion in the opposite (anti-preferred) direction prevents spike generation. Furthermore, we tested the location and the spatial extent of the receptive field of the recorded cells with a small-field pattern moved by hand to exclude other types of spiking motion-sensitive cells. The sampling rate was 20 kHz and spikes were detected offline. The average temperature measured near the thorax of the animals between the recordings was 28.1°C (sd ±1.2°C). Although some influences of temperature on response latency are known (Warzecha et al., 1999), the variance in experimental temperature within FliMax did not affect our conclusions, as it varied much less in given experiments than during different experiments as a consequence of general climatic conditions in the non-air-conditioned laboratory. Moreover, the timescale of response modulations evoked during translatory motion by environmental structures, are relatively slow compared with the temperature-dependent latency changes between approximately 15 and 25 ms. We recorded the response of the H1 cell in seven flies in the experiments with the ‘environment information stimulus’ (overall number of response traces per stimulus condition between 109 to 116, number of response traces per stimulus condition and cell between eight and 27) and in eight animals in the experiments with the ‘adaptation stimulus’ (overall number of response traces per stimulus condition between 123 to 131, number of response traces per stimulus condition and cell between nine and 28).

### Data analysis

#### Stimulus properties

**Fig. 3.**
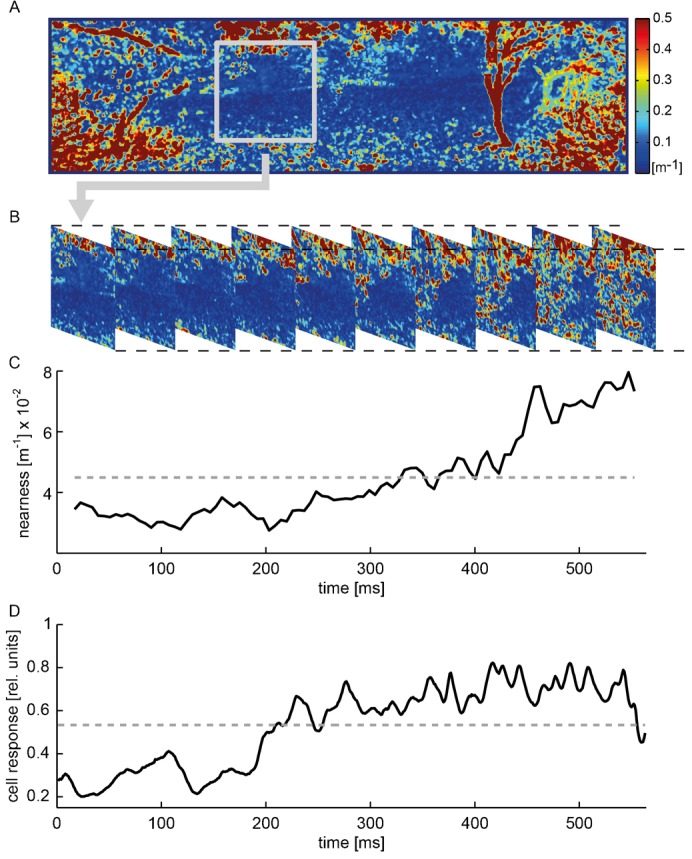
Time course of the nearness and comparison to the cell response during the translation sequence of an example natural scene (see also Fig. 1B). (A) Color-coded panoramic nearness map showing local nearness values. Analysis window depicted as a grey square. (B) Sections of the nearness map showing the changing local nearness values within the analysis window during the translation sequence. (C) Time course of the ‘time-dependent nearness’ averaged across the analysis window (black solid line) and time-averaged nearness (grey dashed line). (D) Time course of the cell response of a single cell (black solid line), normalized to the response to the characterizing stimulus, averaged across repetitions of the translation sequence and average cell response during translation sequence (grey dashed line). Cell response sampled at 200 Hz.

For relating neural responses during simulated self-motion in natural environments to the depth structure of these environments parallax cues from subsequent images were estimated by using the Lucas-Kanade algorithm (Lucas and Kanade, 1981) on the original images with their high-resolution and high dynamic brightness range. Since the distance between the sites where the images were taken are known (10 mm), the distances to environmental structures can be determined from the parallax cues. The distance values estimated in this way were smoothed by temporal Gaussian windows with a width of five images. As a consequence, we had to exclude two images at the beginning and at the end of the image series. As closer objects lead to larger image velocities and should have a greater impact on the cell response than more distant ones, we did not use the distance for correlation with the neural responses, but the nearness of every pixel as given by the inverse distance. To separately determine the time-dependent nearness for the individual time steps of the translational sequence, we averaged the local nearness values within a square window of 60° edge length that roughly covers the most sensitive part of the H1 cell's receptive field (Fig. 3A–C). The window was centered at an azimuth of 45° left of the frontal viewing direction and at 0° elevation. The time-averaged nearness was calculated by averaging the static time-dependent nearness during the entire period of the translational sequence.

We calculated the root-mean-square (RMS) contrast for every image within this analysis window, i.e. the standard deviation of the brightness values of each pixel from the mean brightness, divided by the mean brightness (van der Schaaf and van Hateren, 1996; Brinkworth and O'Carroll, 2009). The RMS contrast was determined after brightness transformation of the images and the projection onto the LEDs of FliMax. Similar to the nearness, we calculated both a ‘time-dependent RMS contrast’ for single images and its time average for the whole translation sequence.

#### Cell responses

Spikes were detected using a threshold that was manually adjusted for each recording. We only used recordings with spike amplitudes at least twice as big as the noise band. Threshold crossings within a time interval of 1 ms after a detected spike were discarded. Spike frequencies were calculated based on the inverse of the duration of inter-spike intervals. Before averaging the responses of different cells to the same stimulus, the responses were normalized to the average spike frequency induced by the characterizing stimulus calculated across a period of 800 ms during motion in the preferred direction. Our main interest was to analyze the responses of H1 cells during simulated translation in natural environments. For the response analysis we took the known average time lag of 20 ms between stimulation and cell response into account (e.g. Warzecha et al., 1999). To examine the relation between the cell response and the stimulus parameters we recorded the cell responses, while presenting the ‘environment information stimuli’. For data analysis we selected 100 time bins within which the cell responses were averaged. Moreover, data across the first to the sixth translation sequence were pooled, as we found no substantial differences between the responses to stimulation with the ‘environment information stimuli’.

To analyze how motion adaptation affects the cell response during the translation sequences, we discretized the cell responses into 5 ms bins. The responses were averaged across each translation sequence separately for cells and stimuli. For assessing the impact of adaptation we divided this result by the response values obtained for the first translation sequence.

Furthermore, we intended to examine the influence of motion adaptation on ‘time-dependent response modulations’ (TDRMs) evoked by environmental properties. Translation through natural sceneries as used in this study led to TDRMs, even if the image velocity within the receptive field of the cell was constant. The TDRMs differed tremendously in their strength between environments depending on their spatial structure and textural properties. Small TDRMs are difficult to quantify with a limited amount of cell recordings, as they could not reliably be distinguished from the random neural response variability (‘response noise’). Therefore, we selected two scenes that evoked much stronger TDRMs than the random modulations of the cell response. The TDRMs evoked during translation sequences were quantified as the standard deviation of the time-dependent response averaged across stimulus repetitions. TDRMs were separately determined for each H1 cell and for each natural scene. The response noise was quantified by calculating the standard deviation of individual response traces of a cell from the corresponding response averaged across the stimulus repetitions. The response noise calculated for individual responses in this way was then averaged across the recordings of each cell. TDRMs were normalized to the average cell response to the corresponding translation sequence. To assess the adaptation effects on the TDRMs we also normalized them to the TDRM obtained for the first translation sequence (‘normalized TDRM’).

## RESULTS

### Influence of nearness and contrast on neural response modulations

While moving through a stationary environment, animals experience motion of the retinal image, which is referred to as optic flow. The optic flow induced by translational self-motion does not only depend on one's velocity, but also on the distance of environmental structures: near objects result in larger angular velocities on the retina than distant ones, and the angular velocities converge to zero for very far distances. However, retinal velocity is just one stimulus parameter that influences the responses of LPTCs: brightness contrast and the specific textural features of environmental structures, such as their spatial frequency content, affect LPTC responses as well. During translatory movements through natural environments both the average nearness of structures in the environment as well as the contrast within the confines of a cell's receptive field may vary continually, depending on the overall three-dimensional layout and the textural properties of the sceneries. To relate the responses of the H1 cell to these environmental features we quantified these stimulus features (Fig. 3): the average nearness and root-mean-square (RMS) contrast were determined within an analysis window and computed as a function of time (‘time-dependent nearness’ and ‘time-dependent contrast’). The window covered the most sensitive part of the H1 cell's receptive field.

**Fig. 4.**
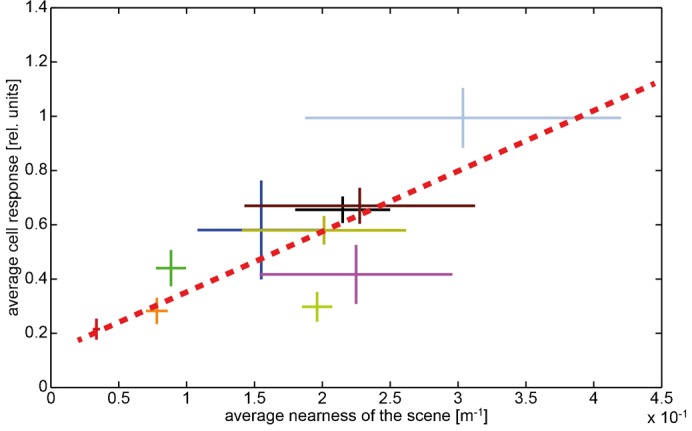
Dependence of the average cell response on the time-averaged nearness for the different natural sceneries. Data obtained in different environments are indicated by different colors. Horizontal bars: standard deviation of the ‘time-dependent nearness’ during the translation sequence within a given scenery. Vertical bars: standard deviation of response modulations obtained during the translation sequence in a given scenery. Corresponding mean values are given by the crossing of the horizontal and vertical bars. Regression line (red dashed line) illustrating the relation between nearness values and cell responses.

The average nearness obtained for the different scenes that we used for stimulation varied between 0.034 m^−1^ and 0.30 m^−1^ (Fig. 4). However, nearness values smaller than 0.033 m^−1^, which correspond to distances of more than 30 m, could not be inferred well by determining the parallax information with the Lucas–Kanade algorithm (see Materials and Methods) because far distant objects only result in very small angular velocities within the optic flow field. Moreover, since near objects in natural scenes often covered only parts of the receptive field, the average nearness within our analysis window is usually smaller than the nearness of the closest object. For instance, a scene with thick trunks of trees (Fig. 1A,F) with a local nearness of 2 m^−1^ only led to an average nearness of below 0.7 m^−1^ within the analysis window. In our set of different natural scenes the variance of the average nearness across the translational sequences differed. It was larger for sceneries with large average nearnesses than for those with small ones (Fig. 4, horizontal error bars).

We examined the relation between the nearness and the response of the cell in two ways: first, we compared the time-averaged nearness with the corresponding averaged cell responses for the translation sequences of one meter length in the different natural scenes (Fig. 4). We found a positive correlation between nearness and neural responses (r^2^ = 0.629). This finding supports our hypothesis that the response of some LPTCs provides at least coarse spatial information about natural environments.

In a second approach, we scrutinized the relation between the time-dependent nearness and the cell response separated in 100 time bins during the translation sequence within individual environments (data not shown). Even within a single environment the overall nearness within the cell's receptive field changes over time depending on the spatial and textural properties of the environment. Accordingly, the neural response may be modulated as a consequence of motion parallax information, despite the translation velocity is constant. However, in a cell with a large receptive field, like the H1 cell, nearness-dependent response changes can be detected only if the average nearness varies sufficiently during a given translatory movement track.

**Fig. 5.**
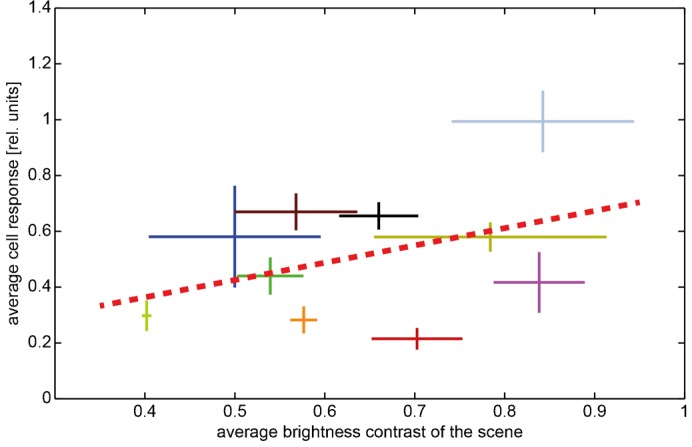
Dependence of the average cell response on the time-averaged RMS contrast of different natural scenes. Data obtained in different environments are indicated by different colors; scenes are specified by the same colors as in Fig. 4. Horizontal bars: standard deviation of the ‘time-dependent RMScontrast’ across the translation sequence in a given scenery. Vertical bars: standard deviation across time of response modulations obtained during the translation sequence in a given scenery. Corresponding mean values are given by the crossing of the horizontal and vertical bars. Regression line (red dashed line) illustrating the relation between contrast values and cell responses.

Another parameter that is known to strongly influence the cell signal, at least when probed with artificial stimuli such as grating patterns, is the brightness contrast (Hausen, 1982; Egelhaaf and Borst, 1989; Hausen and Egelhaaf, 1989; Kurtz et al., 2009). Therefore, we also analyzed the relations between the contrast of the scene and the cell response during a translation sequence (Fig. 5). The RMS contrast was calculated within the analysis window. We did not find any significant dependence of the neural responses on the average RMS contrast during translation sequences in the different natural environments (r^2^ = 0.158) (Fig. 5). We neither detected any indication that the cell was affected by the time-dependent RMS contrast within individual scenes (data not shown). Thus, neither within the scenes nor across the scenes we found any convincing evidence that the RMS contrast of natural scenes was influencing the cell response in an obvious way. This is in line with the results of previous experiments in which pure rotation of natural scenes was used for stimulation (Straw et al., 2008; Barnett et al., 2010). Nevertheless, modulations of the cell signal depending on the environmental layout are clearly visible during the translational stimulation (Fig. 3D).

### Influence of motion adaptation on time-dependent response modulations of the cell

**Fig. 6.**
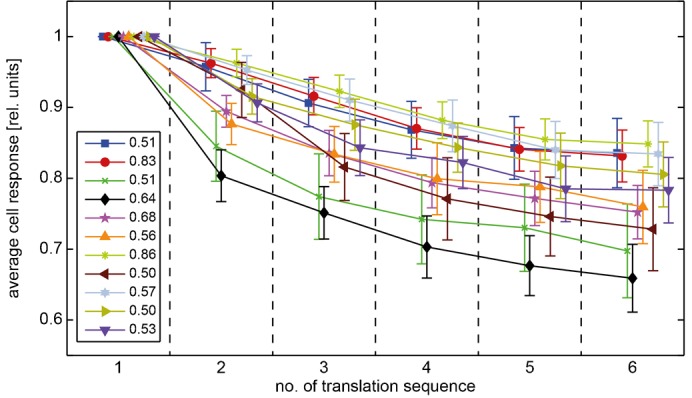
Motion adaptation reduces the average cell response to the translation sequence over time. Data obtained for the different natural scenes are indicated by different colors; colors specify different scenes compared to previous figures. The cell response is time-averaged across single translation sequences and normalized by the value obtained for the first translation sequence (values of the first translation sequence specified in the figure inset, error bars: standard error of the mean across cells). Time is indicated as the number of the translation sequence and the markers of single scenes are slightly shifted along the *x*-axis to enhance visibility.

Motion adaptation is known to strongly affect the response properties of LPTCs. Maintained motion stimulation leads to a decrease of the cell response. In contrast, response modulations evoked by rapid changes in velocity, contrast and motion direction, like those introduced by objects near the flight path of flies, are enhanced by motion adaptation relatively to the overall response level (Maddess and Laughlin, 1985; Heitwerth et al., 2005; Kurtz et al., 2009; Liang et al., 2008; Liang et al., 2011). Since we used natural image sequences with a wide range of depth structures for stimulation, we were able to examine the effects of motion adaptation on response modulations that were evoked by changes in the spatial structure or textural properties of the environment during translational motion. We compared the activation of the cell across six consecutive translation sequences performed in a given environment (Fig. 2) and observed that during translation within natural scenes the average cell responses decreased considerably. However, the extent of this response decline differed between various scenes (Fig. 6).

**Fig. 7.**
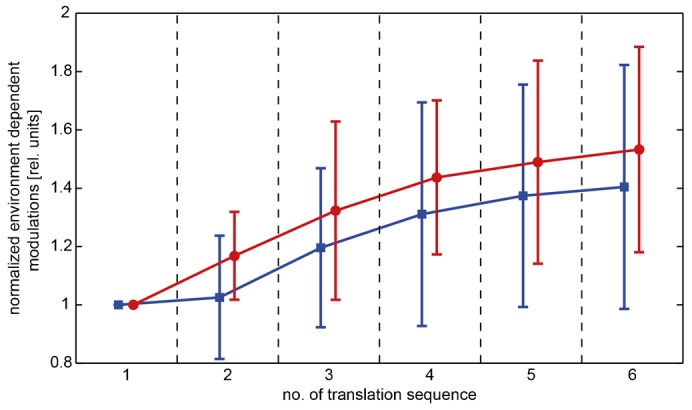
Motion adaptation increases time-dependent response modulations (TDRM) relatively to the average cell response. TDRM normalized to the value obtained for the first translation sequence of two selected scenes (see Materials and Methods), the scenes are plotted in the same color as in Fig. 6. Error bars: standard error of the mean across different cells. Time is indicated as the number of the translation sequence and the markers of single scenes are slightly shifted along the *x*-axis to enhance visibility.

For further investigating how motion adaptation might affect the environment-dependent response modulations we selected two scenes that showed prominent time-dependent response modulations (TDRMs). The response modulations were normalized to the response obtained for the first translation sequence. On average, the standard deviation of the TDRMs increased over the consecutive translation sequences and, thus, increased over time (Fig. 7). This finding indicates that the response modulations depending on the environmental layout are less reduced by motion adaptation than the average response of the H1 cell and, thus, are enhanced relatively to it.

## DISCUSSION

### The influence of nearness

When moving, animals actively facilitate the extraction of information about the three-dimensional layout of their natural environment by generating motion parallax cues. This information can be gathered, processed and then used to guide orientation behavior. Irrespective of brightness, contrast, spatial frequency composition and the phase relations between the different frequency components (e.g. Hyvärinen et al., 2009; Rieke and Rudd, 2009; van der Schaaf and van Hateren, 1996), translational self-motion uncovers the spatial structure of the environment because it induces relative motion in the retinal images of objects located at different distances. The pattern of the velocities in the visual field induced by self-motion is commonly referred to as optic flow (Koenderink, 1986). The influence of distance on the optic flow depends on the type of self-motion: whereas pure rotational motion is independent from the distance, translational motion is affected by the distance of structures in the environment.

The motion sensitive lobula plate tangential cells (LPTCs) of flies (Hausen, 1984; Krapp, 2000; Egelhaaf et al., 2002; Egelhaaf, 2006; Borst et al., 2010) are sensitive to optic flow. As the cell responses depend on image velocity, they might be expected to be affected by the overall distance of surfaces in the environment within the confines of their receptive fields, at least during translatory motion. Previous studies supported this hypothesis by stimulating flies with movies presenting the visual input they would perceive during flight in a box (Kern et al., 2005; van Hateren et al., 2005; Kern et al., 2006; Karmeier et al., 2006; Liang et al., 2008; Liang et al., 2012). While the environments, on which these experiments were based, were artificial and relatively simple, we presented for the first time image sequences obtained during translation in a variety of cluttered natural environments with a wide range of depth structures (Schwegmann et al., 2014). When presenting these stimulus movies, we recorded the response of the H1 cell that is a well explored type of LPTC (Eckert, 1980; Hausen, 1981). We selected the H1 cell just as a typical representative of LPTCs that spatially pool local motion information over extended parts of the visual field and did not intend to address the specific functional role of this cell within the network of LPTCs (Egelhaaf, 2006; Borst et al., 2010). We analyzed the dependency of the cell response on the overall nearness of objects in natural scenes as averaged over an area in the visual field, matching the most sensitive region of the cell's receptive field. A strong correlation between the cell response and the average nearness of the different scenes was found. This result supports the hypothesis that the cell response provides information about the spatial structure of the environment, at least on a coarse spatial scale according to the cell's large receptive field. However, within single natural scenes such dependency was only found in few environments. This result can be attributed to several reasons: (1) H1 integrates visual motion inputs across its large receptive field (Hausen, 1976; Eckert, 1980; Krapp et al., 2001). Thus, changes of a stimulus parameter in solely a small part of the receptive field might affect the cell response only weakly. (2) For the image movies employed in the present study the average nearness varied more between different scenes than within most given scenes. Hence, spatially restricted image features like those introducing high local contrast might have a stronger influence on the cell response, as they may vary more than the average nearness within a given stimulus movie. Based on model simulations, we predict much stronger nearness effects on the neural responses for cells with smaller receptive fields (Schwegmann et al., 2014).

### Contrast dependence in natural scenes

Using natural images for stimulation, we found no significant dependence of the neural response on the RMS contrast within the analysis window. This was the case both for the comparison across the scenes and individual scenes, which is in line with previous studies using natural images without depth structure as motion stimuli (Straw et al., 2008; Barnett et al., 2010). This finding obtained with natural scenes seems to be in conflict with results obtained by varying the contrast of grating patterns. It is here where the responses of LPTCs increased at low Michelson contrasts and saturated at a level of approximately 20% (Dvorak et al., 1980; Egelhaaf and Borst, 1989). Even more confusing, Straw et al. found that a downscaling of the contrast of natural images reduced the response of the cell (Straw et al., 2008).

An explanation of these somehow contradictory results might be led back to the differences between the natural and the experimenter-defined stimuli as well as the significance of different definitions of contrast when applied to natural images and to downscaled versions of them. Whereas the brightness contrast of sine-wave gratings is globally the same across the entire stimulus pattern, the contrasts of natural images may vary tremendously within a given scene (Ruderman and Bialek, 1994; van der Schaaf and van Hateren, 1996; Bex and Makous, 2002; Schwegmann et al., 2014). Features of high local contrast can be found in most natural images, irrespective of the global RMS contrast (Schwegmann et al., 2014). Hence, the global RMS contrast across the entire image might only be a poor predictor of the contrast of local features. As a consequence of the gain control properties of LPTCs, these high-contrast features might have a particularly large influence on the cell response. Gain control limits the influence of contrast and stimulus size on the cell response, while preserving its sensitivity to velocity (Hausen, 1982; Haag et al., 1992; Borst et al., 1995; Single et al., 1997; Grewe et al., 2006). As a result, only few high-contrast image features that move through the receptive field of LPTCs might suffice to reach a large response level, irrespective of the global RMS contrast of a natural image. Hence, the responses of LPTCs to natural scenes can be expected to be largely independent of the global root-mean-square contrast as long as the scenes contain a sufficient number of high-contrast features. Since also the high-contrast image features of natural images are scaled down by reducing the global contrast, it is not surprising that the response amplitude of LPTCs depends on global contrast after this kind of image manipulation (Straw et al., 2008) in a similar way as it depends on contrast variations of grating patterns (Dvorak et al., 1980; Egelhaaf and Borst, 1989). This explanation of the characteristic contrast dependence of LPTC responses when stimulated with natural scenes may still be too simplistic. There is good evidence that the responses of LPTCs are additionally affected by a variety of other non-linearities and local adaptive mechanisms that affect the contrast gain of the cell (Harris et al., 2000; Kurtz et al., 2009; Barnett et al., 2010; O'Carroll et al., 2011; Nordström et al., 2011; O'Carroll et al., 2012; Kurtz, 2012). The potential significance of such adaptive mechanisms for the contrast dependence of LPTC responses to natural image sequences needs to be analyzed in further experiments.

### Motion adaptation

Motion adaptation in LPTCs is known to decrease the cell response to stimulus motion (Maddess and Laughlin, 1985; Harris et al., 2000; Kurtz et al., 2009). This effect was consistently found using experimenter-defined stimuli. By stimulating the cell with natural images rotating around the animal, adaptation had different effects on a short timescale (Barnett et al., 2010): immediately after motion onset, motion adaptation is suggested to reduce the scene-dependent response difference, since it decreased the response to those scenes strongly depolarizing the cell, while it increased the response to scenes only inducing small depolarizations. However, on a longer timescale motion adaptation was found to reduce the responses evoked by strongly depolarizing scenes, but did not affect the response to scenes only weakly depolarizing the cell. It was attributed to the brightness contrast of the scene whether a natural scene induced strong or weak depolarizations (Barnett et al., 2010).

In our study we found a substantial reduction of the cell response to continuous motion stimulation during the consecutive translation sequences in natural environments with depth structure. This was true for all tested natural scenes, including scenes with different contrasts as well as scenes that led to a strong or weak depolarization of the cell. Due to the stimulus design we quantified the effects of motion adaptation on a large timescale between the repeated presentations of the translation sequence that were separated by about 850 ms. Moreover, since we used the optic flow generated during translational motion in cluttered natural environments, the retinal angular velocity varied tremendously within the visual field as a consequence of both, the depth structure of the environment and the retinal location. Thus, our stimulus cannot be characterized by one global angular velocity.

In accordance with previous experiments based on more artificial motion sequences (Maddess and Laughlin, 1985; Kurtz et al., 2009; Liang et al., 2008; Liang et al., 2011), our results suggest that response changes evoked by discontinuities of the stimulus parameters, like transient changes of contrast, velocity or motion direction, are accentuated by adaptation. They either increase during adaptation or are only slightly attenuated and, thus, enhance relatively to the overall activity of the cell because of the pronounced decline in the tonic response level. This finding was interpreted as a strengthening of the responses to stimulus changes as may be induced by objects during flight (Liang et al., 2008; Liang et al., 2011). In line with these results, we found, as a consequence of motion adaptation, a similar relative increase of the response modulations that were induced by the structure of the environment during translational self-motion in cluttered natural environments.

### Conclusions

The results of this study support the hypothesis that LPTCs, like the H1 cell, that pool the output of local motion information over even extended parts of the visual field are able to contribute to the perception of the environmental layout in flies, at least on a coarse spatial scale. In our experiments we examined the influence of environmental visual information on the response of the H1 cell because it can be recorded for sufficiently long times. However, it is very likely that the conclusions can be generalized and also applied to other LPTCs differing in their preferred directions and receptive field location, since LPTCs can generally be assumed to receive input from the same type of local motion detectors and share similar spatial integration properties (e.g. Egelhaaf and Borst, 1993). Nonetheless, further experiments should investigate whether this expectation is correct during self-motion of flies in natural environments.
